# Emergence of clonal evolution with Philadelphia chromosome in acute myeloid leukemia after hypomethylation agents and BCL2 inhibitor treatment

**DOI:** 10.1002/kjm2.12886

**Published:** 2024-08-19

**Authors:** Shih‐Yu Kao, Samuel Y. Hsiao, Bi‐Hua Du, Hui‐Hua Hsiao

**Affiliations:** ^1^ Cancer Center Kaohsiung Medical University Hospital Kaohsiung Taiwan; ^2^ Sidney Kimmel Medical College Thomas Jefferson University Philadelphia Pennsylvania USA; ^3^ Hematology‐Oncology Department Kaohsiung Medical University Hospital Kaohsiung Taiwan; ^4^ Faculty of Medicine Kaohsiung Medical University Kaohsiung Taiwan

The Philadelphia chromosome (Ph) resulting from translocation of t(9;22) which generate BCR::ABL fusion oncogene is mostly happened in chronic myeloid leukemia and acute lymphoblastic leukemia, but rarely seemed in acute myeloid leukemia (AML).^1^ Clonal evolution with Ph+ chromosome arising from the treatment of AML lacking Ph+ clone/BCR:ABL has rarely been reported.^2^, ^3^, ^4^ Here we report two rare cases of acquisition Ph+/BCR::ABL fusion after hypomethylation agent (HMA) and BCL2 inhibitor therapy.

## CASE 1

A 67‐years‐old female, diagnosed as AML with *FLT3‐ITD* mutation in February 2017, received four courses of low dose ara‐c (LDAC) with response. She had azacitidine therapy (100 mg × 5 days every 28 days) for maintain, but disease relapsed in January 2018. Venetoclax (200 mg qd) was added in combination with azacytidine due to poor ECOG status. In that time, normal Karyotyes without BCR::ABL transcript nor FLT‐ITD mutations were noted (Table 1). However, poor response with clonal evolution of Ph+ abnormality and BCR::ABL was detected in March 2018. The patient expired 1 months later due to refractory of AML.

**TABLE 1:** Patients' status.

Age/gender	Diagnosis date	Initial mutations	Initial cytogenetic	Induction therapy	Cytogenetic study before HMA/BCL‐2	BCR::ABL	Treatment	Cytogenetic after HM*A/*BCL2	BCR::ABL
67/F	2017/02	FLT3‐ITD(+), NPM1(−), AML1‐ETO(−), CBFB‐MYH (−)	48, +6, +8[1]/46, XX[20]	LDARAC for 2 courses, HMA	46, XX[23]	–	AZA + VEN	t(9;22)(q34;q11)[10]/46, XX[12]	P230
66/F	2021/06	NGS study: normal	46, XX[25]	I3A7, HDARAC	46, XX[23]	–	AZA + VEN	t(9;22)(q34;q11) [3]/46, XX[22]	P230

## CASE 2

A 66‐years‐old female, diagnosed as AML in June 2021, received I3A7 (Idarubicine + cytarabine) regimen followed by two courses of high dose cytarabine therapy with remission achieved. Normal karyotypes without molecular abnormalities by NGS were noted then. She had azacytidine (100 mg × 5 days) + venetoclax for maintain therapy since March 2022. However, relapse of AML with emergence of Ph+ clone and BCR::ABL transcription was noted in August 2022. The patient expired 2 months later with refractory to imatinib and chemotherapy.

Though rare, our results contributed to the understanding of Ph+ clone/BCR::ABL fusion transcript in the pathomechanism AML evolution. For AML harboring Ph+ clone/BCR::ABL transcript, which generally carries poor outcomes,^3^, ^4^, ^5^ is been proposed by WHO/ELN recommendation as a distinct entity and might benefit from TKIs combined with chemotherapy. Our cases, with emergence of acquisition Ph+/BCR::ABL shortly after HMA and BCL2 inhibitor, implies this evolution might contribute to disease progression and poor prognosis. With limited cases, our patients with poor outcomes suggest that this pathomechanism might be resistant to salvage treatment and warrant further investigate of novel strategies to rescue these kinds of patient.

## CONFLICT OF INTEREST STATEMENT

The authors declare no conflict of interest.

## References

[1] Soupir CP , Vergilio JA , Dal Cin P , Muzikansky A , Kantarjian H , Jones D , et al. Philadelphia chromosome‐positive acute myeloid leukemia: a rare aggressive leukemia with clinical pathologic features distinct from chronic myeloid leukemia in myeloid blast crisis. Am J Clin Pathol. 2007;127:642–650. 17369142 10.1309/B4NVER1AJJ84CTUU

[2] Rosenberg MW , Savage SL , Eide CA , Reister Schultz A , Cook RJ , Press RD , et al. Comprehensive molecular characterization of a rare case of Philadelphia chromosome‐positive acute myeloid leukemia. Cold Spring Harb Mol Case Stud. 2022;8:a006218. 36307214 10.1101/mcs.a006218 PMC9632359

[3] Alotaibi AS , Yilmaz M , Loghavi S , DiNardo C , Borthakur G , Kadia TM , et al. Emergence of BCR‐ABL1 fusion in AML post FLT3 inhibitor‐based therapy: a potentially targetable mechanism of resistance—a case series. Front Oncol. 2020;10:a588876. 10.3389/fonc.2020.588876 PMC7606916 33194747

[4] Kurt H , Zheng L , Kantarjian HM , Tang G , Ravandi‐Kashani F , Garcia‐Manero G , et al. Secondary Philadelphia chromosome acquired during therapy of acute leukemia and myelodysplasia syndrome. Mod Pathol. 2018;31:1141–1154. 29449681 10.1038/s41379-018-0014-x

[5] Keung YK , Beaty M , Powell BL , Molnar I , Buss D , Pettenati M , et al. Philadelphia chromosome positive myelodysplastic syndrome and acute myeloid leukemia—retrospective study and review of literature. Leuk Res. 2004;28:579–586. 15120934 10.1016/j.leukres.2003.10.027

